# Validity and Reliability of a Korean Version of the ConCom Safety Management Scale

**DOI:** 10.3390/ijerph182312514

**Published:** 2021-11-27

**Authors:** Mi Young Kwon, Nam Yi Kim

**Affiliations:** 1 Department of Nursing, Gimcheon University, Gimcheon 39528, Korea; mykwon82@gmail.com; 2 College of Nursing, Konyang University, Daejeon 35365, Korea

**Keywords:** nursing care, nursing, patient safety, safety management, organization and administration

## Abstract

The purpose of this study was to verify the validity and reliability of the Korean version of the ConCom Safety Management Scale (K-CCSMS). This study consisted of two phases. First, in accordance with the guidelines of the World Health Organization, the Korean version of the scale was developed in five stages. Second, data from 206 general and tertiary hospital nurses were analyzed to confirm the validity and reliability of the K-CCSMS; thus, the construct validity, criterion-related validity, and reliability were confirmed. In total, 21 items divided across four factors (i.e., stressing the importance of safety rules and monitoring, providing employees with feedback, showing role modeling behavior, and creating safety awareness) were identified through exploratory factor analysis. Three items were deleted through confirmatory factor analysis, and the model fit was as follows: normed χ^2^ = 2.80, normed fit index = 0.87, Tucker–Lewis index = 0.90, comparative fit index = 0.92, and standardized root mean square residual = 0.05. The correlation coefficient between the K-CCSMS and patient safety culture was 0.76 (*p* < 0.001), and internal consistency was acceptable (Cronbach’s α = 0.95). For patient safety, an appropriate combination of control- and commitment-based management is required, and the 18-item K-CCSMS showed usefulness and reliability in determining such a balance and evaluating the leadership styles of Korean nursing managers.

## 1. Introduction

Nurses play a pivotal role in patient safety and are instrumental in the early discovery of errors and harm prevention [1]. Thus, safety leadership in nurses is considered an important factor in improving and ensuring patient safety in hospitals [2]. In particular, nurse managers develop and provide patient safety guidelines [3], stress the importance of patient safety such that all nurses in the organization can participate, serve as role models, and encourage participation in activities related to patient safety management [4]. Therefore, nurse managers’ leadership is crucial, as it fosters a patient-safety-promoting environment in the ward and facilitates improvement activities.

Organizations must promote various leadership behaviors among managers and implement management measures to meet patient safety goals [5]. In general, studies on human resource management report two types of management approaches: control-based management and commitment-based management [6]. Control-based management is a typical top-down approach that focuses on regulating, supervising, and controlling the behaviors of the members of an organization [7]. Commitment-based management increases awareness of the mission, vision, and objectives of the organization and facilitates the internalization of patient safety regulations and values among members [7].

Patient safety is the protection of patients from harm. Receiving safe treatment in a safe environment is one of patients’ fundamental rights, as the Joint Commission International prioritizes improving patient safety and focuses on all evaluation items [8]. For example, in general, strict safety regulations are enforced at nuclear power plants, military bases, and airports. If the employees therein fail to comply with the regulations, through control-based management, managers will apply disciplinary action to improve compliance [9]. Prior to the establishment of a method for patient safety management, hospitals typically employed safety management regulations used by public institutions; however, this failed to improve employee compliance [10]. This is because applying control-based management to patient safety—which, compared to other safety categories, is characterized by higher levels of uncertainty, variability, and complexity—causes difficulties due to specific hospital characteristics such as clinical autonomy and non-hierarchical working relationships [4]. Nursing managers’ compliance with safety rules and improvement of subjective standards—through monitoring and control of organizational members—is essential for effective patient safety management [11]. However, it is difficult to increase employees’ safety management performance without commitment-based management, which promotes the intrinsic motivation to ensure patient safety in dynamic hospital situations [12]. In other words, both the control-based approach, which includes rules and regulations, and the commitment-based approach, which can facilitate internal motivation, are considered important. Specifically, since nurse managers play a key role in safely caring for patients through close interactions with other members of the organization, the two management approaches should be implemented in a complementary manner, as opposed to exclusively using one of them [4].

Thus far, in Korea, there are no scales for measuring the effectiveness of both control- and commitment-based management in an integrated and specific manner. Currently, the Perception of Importance on Patient Safety scale is the most commonly used tool in Korea to measure employees’ awareness of patient safety management [13]. Alingh et al. developed the ConCom Safety Management Scale (CCSMS) to measure employees’ perception of mutually complementary management by integrating control- and commitment-based management [14]. The CCSMS integrates control- and commitment-based management approaches and focuses on nurse managers’ leadership behaviors regarding patient safety management in hospitals [4]. Since organizational culture reflects a country’s unique characteristics, it may not be appropriate to use scales from other countries in their original form [15]: adaptation is required. However, adapting a scale goes beyond simply translating it. For example, words with the same dictionary meaning used in a scale may apply differently depending on the cultural context. In other words, although scales can be used in a context different from the one within which they were developed, linguistic, idiomatic, contextual, and cultural aspects should be considered in the transcultural adaptation of a scale. By so doing, a scale developed in Germany can be translated, adapted, and validated for use in Korea [11]. Thus, the purpose of this study was to establish the validity and reliability of the Korean version of the ConCom Safety Management Scale (K-CCSMS) for obtaining information about the degree of control- and commitment-based safety management approaches employed by Korean nurse managers and improving patient safety.

## 2. Materials and Methods

### 2.1. Study Design

This was a methodological study for adapting the CCSMS [14] for the Korean context and testing that version’s validity and reliability. The scale was first translated into Korean considering Korea’s clinical context and then assessed for content validity by nursing experts in patient safety management. A cross-sectional survey was subsequently conducted with clinical nurses.

### 2.2. Sample

The study comprised a conveniently sampled cohort of 206 nurses directly involved in patient care at general or tertiary hospitals with 500 beds or more in the cities S, P, or D.

### 2.3. Instruments

The participating nurses completed a questionnaire comprising three sections: (1) participant’s demographics, (2) CCSMS, and (3) patient safety culture.

#### 2.3.1. Participant’s Demographics

Information was collected about participants’ age, gender, marital status, education level, clinical career, unit, experience with patient safety incidents, and completion of education related to patient safety incidents in the past 6 months.

#### 2.3.2. CCSMS

The original is a 33-item scale that examines nurses’ perceptions of their managers’ control- and commitment-based patient safety management approaches [14]. The scale comprises seven subscales: stressing the importance of safety rules and regulations (5 items), monitoring compliance (4 items), providing employees with feedback (3 items), showing role modeling behavior (7 items), creating safety awareness (6 items), showing safety commitment (5 items), and encouraging participation (3 items). The five items in the “stressing the importance of safety rules and regulations” subscales are rated on a 4-point scale, and the rest are rated on a 5-point scale. The 5-point scale ranges from 1 (never true) to 5 (always true), and the 4-point scale ranges from 1 (never true) to 4 (always true), with no neutral rating. The final score is calculated by converting the 4- and 5-point scale scores to an equivalent scale. The total score ranges from 33 to 165, with a higher score indicating stronger control- and commitment-based safety management. The internal consistency of the scale (Cronbach’s α) at the time of development was 0.70 for stressing the importance of safety rules and regulations, 0.59 for monitoring compliance, 0.64 for providing employees with feedback, 0.90 for showing role modeling behavior, 0.86 for creating safety awareness, 0.90 for showing safety commitment, and 0.82 for encouraging participation [14].

#### 2.3.3. Patient Safety Culture

An organization’s patient safety culture is correlated with the types of leadership and can predict safety management activities [16]. Thus, it was chosen as the construct for the criterion validity assessment. For this, we used the patient safety culture scale, developed by Lee [13], which comprises organization, department, and individual domains. It comprises 35 items in total. Each item is rated on a 5-point Likert scale ranging from 1 (never true) to 5 (always true), with a higher score indicating a higher perception of patient safety culture. The internal consistency (Cronbach’s α) of the scale was 0.93 at the time of development [13] and 0.94 in this study.

### 2.4. Data Collection

The sample size was set to 220 in accordance with the standard that the sample size should be 5–10 times larger than the number of items and larger than 200 for structural equational modeling estimation [17,18]. We also set the sample considering a 10% withdrawal rate. After excluding 6 nurses who withdrew from the study and 8 who did not respond to more than 10% of the questionnaire, data from 206 nurses were included in the final analysis. Data were collected from 25 May 2020 to 30 June 2020.

### 2.5. Data Analysis

The collected data were analyzed using SPSS (version 25.0; IBM, Armonk, NY, USA) and AMOS version 21.0; IBM, Chicago, IL, USA). Participants’ demographics were analyzed using descriptive statistics and the normality of the data was tested. Construct validity was tested using item analysis with correlation coefficients, exploratory factor analysis (EFA), and confirmatory factor analysis (CFA). The fit of the model was tested based on χ^2^, normed χ^2^ (χ^2^/df), comparative fit index (CFI), Tucker–Lewis index (TLI), root mean square error of approximation (RMSEA) and standardized root mean square residual (SRMR) [19]. Because χ^2^ is sensitive to the complexity of a model and/or sample size, a χ^2^ value is not automatically calculated by the statistical program, but is calculated by manual calculation. Further, it has been confirmed that this value is not presented separately in previous studies. A χ^2^/df value of 3 or lower is deemed appropriate; moreover TLI and CFI values of 0.90 or higher and RMSEA and SRMR of 0.08 or lower are considered to indicate a good fit [19,20]. In the present study, the TLI values did not meet the cutoff for goodness of fit. The standardized regression weights (SRWs) for establishing convergent validity must be 0.50 or higher and a value of 0.70 or higher is desirable [20]. The convergent validity of the items was established by examining factor loadings, construct reliability (CR), and average variance extracted (AVE), while discriminant validity was verified using correlation coefficients and coefficients of determination. Criterion validity was analyzed based on the correlation with patient safety culture using Pearson’s correlation.

## 3. Results

### 3.1. Results of the Translation-Adaptation Process

In accordance with the World Health Organization guidelines for scale translation and adaptation [15], the CCSMS was translated and adapted into Korean in five stages.

#### 3.1.1. Forward Translation

Prior to translation, we obtained permission to use the scale from the original developer (Alingh). The forward translation was performed by two nursing Ph.D. holders with English fluency and a clinical career of at least 15 years, and the translations were compared to develop the Korean translation.

#### 3.1.2. Expert Panel Back Translation

An expert panel comprising patient safety nursing experts compared the translation with the original scale and presented opinions about inadequate expressions and concepts. The panel specifically consisted of six experts: two nursing instructors with experience in nursing research and scale translation, two nursing unit chiefs with a nursing Ph.D. and a clinical career of at least 20 years, and two patient safety management unit chiefs of a hospital with a nursing Ph.D.

The experts confirmed that none of the translated items showed cultural gaps, but two items were revised to better clarify the context. Item 14, “Whenever pressure builds up, my supervisor wants us to work faster, even if it means taking shortcuts”, was revised to “My supervisor wants me to work more quickly even if it means taking a shortcut when the workload increases.” Item 29, “We are given feedback about changes put into place based on event reports”, was revised to “We are given feedback about any changes about patient safety using an incident report.”

The back translation of the revised Korean version was performed by a native English professor with more than 10 years of teaching experience in Korea and an understanding of nursing. The back-translated scale was compared with the original scale by the researcher and translator to check for any changes in meaning. It was agreed that there were no marked differences between the translation and back translation.

#### 3.1.3. Evaluation of Content Validity

The expert panel for content validity testing consisted of five experts: two nursing instructors, two general hospital unit chiefs with a nursing Ph.D., and one patient safety unit chief. The assessment was performed using the content validity index (CVI). Each item was rated on a 4-point scale ranging from 1 (not relevant) to 4 (highly relevant) and the item-level content validity index (I-CVI) and scale-level content validity index (S-CVI) were computed. Items with an I-CVI of 0.83 or higher, a universal agreement S-CVI (S-CVI/UA) of 0.80 or higher, and an average S-CVI (S-CVI/Ave) of 0.90 or higher were deemed relevant [21]. In the first round of evaluation, 12 items did not meet the cutoffs, with an I-CVI of 0.20–0.40; however, the remaining items had an I-CVI of 1.0. The expert panel suggested that these 12 items be deleted, as many of them were redundant. These included, “In this department, it is considered extremely important to follow safety rules and procedures,” “In this department, everything has to be done by the book,” “My supervisor always practices the safety protocols he/she preaches,” and “My supervisor does not actually prioritize safety issues as highly as he/she says he/she does.” Further, only the five items in the “stressing the importance of safety rules and regulations” dimension in the original scale used a 4-point scale; therefore, the expert panel unanimously suggested that the same rating scale be used for the entire scale for convenience of response and statistical analysis. Moreover, adopting a 5-point scale would not lead to significant changes in the responses. Thus, we deleted the non-compliant 12 items, unified the rating scale, and performed a second round of CVI evaluation for the remaining 21 items. All items showed ICV-I, S-CVI/UA, and S-CVI/Ave of 1.00.

#### 3.1.4. Pre-Testing and Cognitive Interviewing

This stage was conducted from 4 May 2020 to 15 May 2020 with nurses who met the inclusion criteria. The appropriate sample size for a pilot test was 20–40; therefore, we recruited 30 nurses [17]. Participants’ opinions were obtained about the clarity of sentences, appropriateness of words, appropriateness of the construct under study, and any difficulties or questions. All participants stated that the word “outcomes” in item 20, “We are generally informed about the patient outcomes available for our department,” should be revised to “information,” as it could suggest that the information is given only after the fact. Thus, the item was revised to “We are generally given information about the outcomes of patients in our unit.”

#### 3.1.5. Finalization of Translation

The 21-item preliminary version of the scale was completed. The final questionnaire consisted of 2 items for stressing the importance of safety rules and regulations, 3 items for monitoring compliance, 3 items for providing employees with feedback, 4 items for showing role modeling behavior, 4 items for creating safety awareness, 3 items for showing safety commitment, and 2 items for encouraging participation.

### 3.2. Participants’ General Characteristics

Nurses’ mean age was 30.9 ± 8.05 years and the majority was 20–29 years old (52.4%). Most participants were female (93.2%) and single (66.5%). The mean length of clinical career was 7.75 ± 7.29 years. Of the participants, 44.6% worked in a general ward and 52.4% had experienced patient safety incidents before. Most participants had completed either one (51.0%) or two (26.7%) patient safety education programs within the past 6 months (Table 1).

### 3.3. Validity Testing

#### 3.3.1. Construct Validity

For the 21 items of the preliminary Korean version, factors were developed using principal component analysis (PCA) and determined with EFA using varimax rotation. Four factors emerged. “Stressing the importance of safety rules and regulations” and “monitoring compliance” were integrated into one factor, “showing role modeling behavior,” “showing safety commitment,” and “encouraging participation” were integrated into another factor, and the other two factors remained the same as in the original.

CFA was performed on the items. The model fit indices were as follows: χ^2^ = 468.73 (*p* < 0.001), χ^2^/df = 2.56, CFI = 0.90, TLI = 0.89, RMSEA = 0.78, SRMR = 0.05 (Table 2).

**Figure 1:**
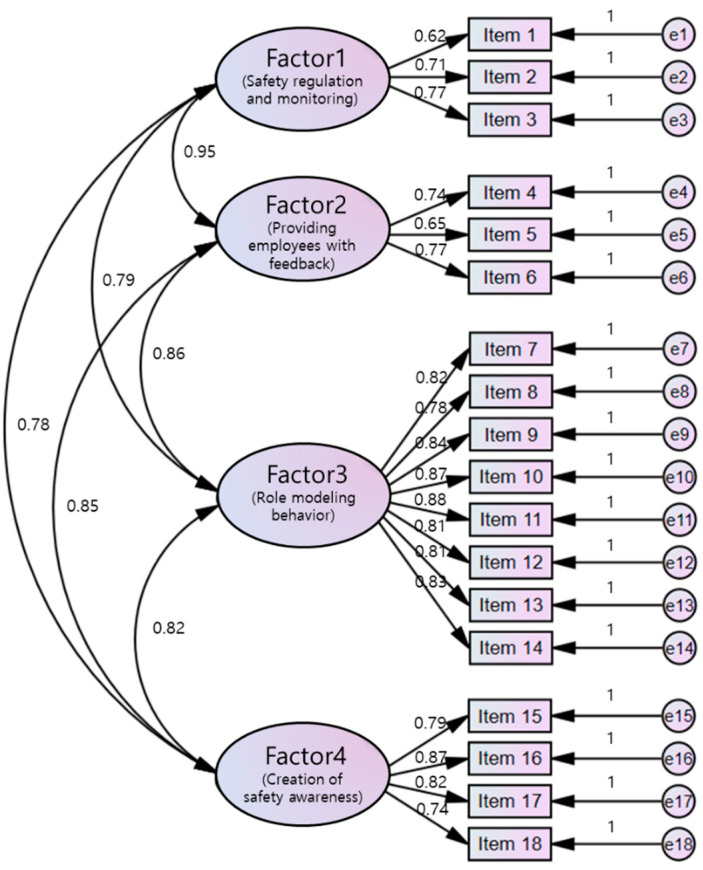
Measurement model for the Korean version of the ConCom Safety Management Scale.

Items 4, 7, and 12 had a SRW of 0.50 or lower and were thus deleted, leaving a total of 18 items in the Korean version. A second CFA performed after deleting three items showed SRWs of 0.62–0.88 (Table 2) (Figure 1). The model fit indices were χ^2^ = 374.29 (*p* < 0.001), χ^2^/df = 2.80, CFI = 0.92, TLI = 0.90, RMSEA = 0.74, and SRMR = 0.05 (Table 2).

The overall fit of the model improved and was deemed relatively good. CR and AVE must be 0.70 or higher and 0.50 or higher, respectively [20], and both satisfied the criteria with a range of 0.72–0.95 and 0.50–0.69, respectively, in this study (Table 3).

Discriminant validity is established when the AVE is greater than the square of the correlation coefficient (r) of each latent variable; that is, the coefficient of determination (r2) [20]. The correlation analysis showed that the coefficient of determination (r2) of each factor was smaller than the AVE, thereby confirming discriminant validity (Table 3).

#### 3.3.2. Criterion Validity

Patient safety management has a positive effect on patient safety culture. To test the criterion validity, we analyzed the correlation between the Korean version and patient safety culture scores. In general, a correlation of 0.40–0.80 is recommended when testing the criterion validity [22] and the results confirmed a correlation coefficient of 0.76 (*p* < 0.001), indicating a significant positive correlation.

#### 3.3.3. Reliability Testing

Cronbach’s α for the four factors of the 18-item Korean version was 0.69 for Factor 1, 0.65 for Factor 2, 0.93 for Factor 3, and 0.88 for Factor 4. That for the entire scale was 0.95.

#### 3.3.4. Finalization of Scale

The K-CCSMS was finalized with 18 items across four factors. Factor 1, which encompassed “stressing the importance of safety rules and regulations” and “monitoring compliance” in the original scale, was labeled “safety regulations and monitoring” (Table 4). Although Factor 3 included “showing safety commitment,” “encouraging participation,” and “showing role modeling behavior” in the original scale, it was labeled “role modeling behavior” because its items mostly measured role modeling behavior.

The finalized 18-item K-CCSMS comprised 3 items for “safety regulations and monitoring,” 3 items for “providing employees with feedback,” 8 items for “role modeling behavior,” and 4 items for “creation of safety awareness.” The scale uses a 5-point scale and the total score ranges from 18 to 90, where a higher score indicates stronger control- and commitment-based safety management.

## 4. Discussion

This study aimed to adapt and validate the CCSMS for use in Korea. First, the scale was translated into Korean. The main survey was conducted using the original scale, followed by statistical construct validity testing to finalize the Korean version.

When translating and adapting a scale with an established construct validity, the scale may be validated only through CFA without EFA [23,24]; however, we performed EFA to determine whether the factor classification differed from that of the original scale. The original scale comprised seven factors [14], but the Korean version was reduced to four factors based on the results of the EFA. In the original scale, the factors “stressing the importance of safety rules and regulations,” “monitoring compliance,” and “providing employees with feedback” were considered to reflect control-based safety management. In this study, we determined that Factor 1 was largely consistent with the original scale in measuring this specific domain; thus, Factor 1 was labeled “safety regulations and monitoring.” However, Item 3, which was included in the original tool’s “monitoring performance,” was separated. Korean nurses regard the meaning of “monitoring” to be a periodic and formal process at the organizational level in accordance with the patient safety management system of the hospital [25]. The “walk rounds” presented as an example of Item 4 means observing an individual and immediately providing feedback concerning the problem discovered. A previous study on Korean nurses identified that the organizational atmosphere that emphasized that it was an individual’s problem rather than a systematic error when problems related to patient safety occurred was common [26]. This idea is also regarded to have affected nurse’s perceptions of “monitoring” and “walk rounds” differently.

We determined that the “showing safety commitments” and “encouraging participation” factors in the original scale featured role modeling behaviors. Further, “showing role modeling behavior” in the original scale was relatively strongly correlated with “showing safety commitments” and “encouraging participation.” Thus, these factors were merged into Factor 3, which was thus labeled “role modeling behavior.”

With regard to patient safety, efforts to provide free questioning opportunities and to promote teamwork are essential [27]. Nevertheless, the larger the hospital, the more important it is to have a formal, disciplined, and hierarchical organizational culture [28]. Korean nurses feel burdened with doing their work quickly within a certain time, so they focus on improving work efficiency rather than exploring new solutions to problems [29]. There is also a widespread perception that nursing managers are solely responsible for patient safety [30]. Based on previous studies, nurses are using modeling nursing managers as a guide for rapid performance in hierarchical organizational settings.

After finalizing the four factors as per the results of the EFA, CFA was performed. TLI did not satisfy the criteria; therefore, the SRWs were examined [31], and three items with inappropriate SRWs were deleted. In this study, the item convergence criterion for the SRW was set to a minimum of 0.50 [31], so a criterion of 0.70 should be used to improve the measurement model. However, in our study, there was one item with a SRW below 0.70, but it was essential for the construct and measurement of the variable. Hence, the item was not removed, and the structure of the scale was retained. Furthermore, when other fit indices were viewed together, the model had a relatively good fit.

In the analysis of correlation coefficients, coefficients of determination, and AVE between the factors, each factor was found to be mutually independent [20]. Although we cannot compare the Korean version directly with the original scale because of the difference in the factors, the correlations between “safety regulations and monitoring” and “providing employees with feedback” (r = 0.65, *p* < 0.001) and between “showing role modeling behavior” and “creation of safety awareness” (r = 0.75, *p* < 0.001) in this study were stronger than those between control-based “monitoring compliance” and “providing employees with feedback” (r = 0.51, *p* < 0.01) and between commitment-based “showing role modeling behavior” and “creating safety awareness” (r = 0.48, *p* < 0.01) in the original scale. The increased correlation seems to be due to the merging of the factors, but the fact that all factors satisfied the criteria for independence shows that the 3 items for “safety regulations and monitoring,” 3 items for “providing employees with feedback” (control-based), 8 items for “role modeling behavior,” and 4 items for “creation of safety awareness” (commitment-based) adequately described the corresponding construct and were distinguished from other factors.

In this study, the internal consistency (Cronbach’s α) of the Korean version was 0.69 for “safety regulations and monitoring,” 0.65 for “providing employees with feedback,” 0.93 for “role modeling behaviors,” and 0.88 for “creation of safety awareness.” In the original scale, Cronbach’s α was 0.70 for “stressing the importance of safety rules and regulations,” 0.59 for “monitoring compliance,” 0.64 for “providing employees with feedback,” 0.90 for “showing role modeling behavior,” 0.86 for “creating safety awareness,” 0.90 for “showing safety commitment,” and 0.82 for “encouraging participation” [14]. Although internal consistency is generally higher with a greater number of items [32], the internal consistency of the K-CCSMS was higher despite having fewer items, confirming that it is a reliable scale.

Based on these results, the validity and reliability of the scale was established. However, many of the original items were deleted; thus, the Korean version has a different structure from that of the original scale, calling for cautious interpretation of results. Nevertheless, this study is significant in that it developed and established the validity and reliability of the CCSMS for use among nurses in Korea. It could thus be used to understand the type of control- and commitment-based safety management approach demonstrated by managers and intervene in patient safety management activities and outcomes.

Three limitations of the study suggest directions to improve the generality and usefulness of the present findings. First, because the present study deleted items from the original scale, future research could test how well results obtained with the full scale match those found here. Second, because the study centered on some regions and nurses in hospitals with 500 beds or more, the generalizability of the scale to other situations may be ambiguous. Thus, future research would do well to test the safety management scale in other regions and among nurses in smaller hospitals. Additionally, further research could increase and re-examine the criteria for convergence, which the present study set to a minimum.

Nevertheless, the adapted scale will contribute to examining the types of patient safety problems to reflect organizational characteristics; coordinating types of leadership demonstrated by nurse managers; and ultimately developing specific strategies to promote patient safety further through research on patient safety culture as well as management activities. K-CCSMS will be instrumental in constructing a theoretical model of the structural relationship between control- and commitment-based management and actual patient safety, which will enable intervention development.

In Korea, a patient safety act was enacted to improve the quality of patient safety. However, most of the content that can be stipulated in the act concerns the obligation to report. As mentioned in this study, in order to improve the quality of patient safety, it is necessary to properly employ both control- and commitment-based management approaches and establish an effective patient safety feedback system. To this end, the K-CCSMS could provide evidence for determining the appropriateness of national patient safety management and establishing standardized patient safety management.

## 5. Conclusions

Patient safety issues are influenced by organizational management types. Hence, an appropriate scale is necessary to assess nurse’s perception of control- and commitment-based patient safety management. Thus, this study aimed to develop and validate the K-CCSMS. The developed version comprised 18 items rated on a 5-point scale, from 1 (never true) to 5 (always true), with a higher score indicating stronger control- and commitment-based leadership. The results showed that the scale had high reliability. The measurement model had a relatively good fit. In addition, content validity and criterion validity were established. The K-CCSMS will be useful in evaluating nurses‘ perceptions of their manager’s control- and commitment-based patient safety management in Korea. The K-CCSMS was investigated by nurses in some hospitals, so it is necessary to establish its feasibility and generalizability in future studies. Further research is recommended to identify the effect of such approaches to patient safety management on patient safety in Korea.

## Figures and Tables

**Table 1:** General characteristics of participants.

Characteristics	Categories	*n* (%)	Mean ± SD ^a^
Age (years)	20–29	108 (52.4)	30.9 ± 8.05
	30–39	65 (31.6)	
	40–49	28 (13.6)	
	≥50	5 (2.4)	
Gender	Male	14 (6.8)	
	Female	192 (93.2)	
Marital status	Unmarried	137 (66.5)	
	Married	69 (33.5)	
Education	College	36 (17.5)	
	Bachelor	147 (71.3)	
	Master	23 (11.2)	
Clinical career (years)	<3	68 (33.0)	7.75 ± 7.29
	3 ~ <5	29 (14.1)	
	5 ~ <10	39 (18.9)	
	≥10	70 (34.0)	
Work department	General ward	92 (44.6)	
	Operation room/Recovery room	36 (17.5)	
	Emergency room	34 (16.5)	
	Intensive care unit	22 (10.7)	
	Other	22 (10.7)	
Patient safety accident experience	Yes	108 (52.4)	
	No	98 (47.6)	
Completion of patient safety education programs	0	27 (13.1)	
	1	105 (51.0)	
	2	55 (26.7)	
	≥3	19 (9.2)	

^a^ Standard deviation. ~ <: more than some years, but less than some years.

**Table 2:** Confirmatory factor analysis of the measurement model.

Model Fit	χ^2^	df ^a^	χ^2^/df	CFI ^b^	TLI ^c^	RMSEA ^d^	SRMR ^e^
	(*p*)						
Model 1	468.73	183	2.56	0.9	89	0.78	0.05
(Item 21)	(*p* < 0.001)						
Model 2	374.29	129	2.8	0.92	0.9	0.74	0.05
(Item 18)	(*p* < 0.001)						

^a^ degrees of freedom; ^b^ comparative fit index; ^c^ Tucker–Lewis index; ^d^ root mean square error of approximation; ^e^ standardized root mean squared residual.

**Table 3:** Correlations between factors and verification of construct validity.

Factors	Factor 1	Factor 2	Factor 3	Factor 4	AVE ^a^	CR ^b^
	r	r	r	r		
	(*p*)	(*p*)	(*p*)	(*p*)		
Factor 1	1				0.50	0.72
Factor 2	0.65	1			0.52	0.76
	(<0.001)					
Factor 3	0.66	0.66	1		0.69	0.95
	(<0.001)	(<0.001)				
Factor 4	0.62	0.63	0.75	1	0.65	0.88
	(<0.001)	(<0.001)	(<0.001)			

^a^ Average variance extracted; ^b^ Construct reliability.

**Table 4:** Dimensions and items in the Korean version of ConCom Safety Management Scale.

Dimension (CCSMS)	Dimension (K-CCSMS)	Item	
Formalization	Safety regulation and monitoring	1	In this department, it is considered extremely important to follow safety rules and procedures (e.g., regarding hand hygiene).
Monitor compliance		2	In this department, it is rarely monitored whether employees comply with safety rules and procedures.
		3	When my supervisor is in the department, he/she monitors whether we comply with safety rules and procedures (e.g., regarding hand hygiene).
	Providing employees with feedback	4	In this department, employees’ compliance with safety rules and procedures is monitored on a regular basis, for example during safety audits or walk rounds.
Provide feedback on (non-)compliance		5	In my department, anyone who violates safety rules or procedures is swiftly corrected.
		6	Compliance with safety rules and procedures (e.g., regarding hand hygiene) does substantially contribute to a positive assessment in our department.
Prioritize patient safety	Role modeling behavior	7	The actions of my supervisor show that patient safety is a top priority.
Show role modeling behavior		8	Regarding safety, my supervisor delivers the consequences he/she describes.
Show commitment on patient safety		9	My supervisor behaves in a way that displays a commitment to patient safety.
		10	My supervisor provides continuous encouragement to do our jobs safely.
		11	My supervisor shows determination to maintain a work environment where we deliver safe care to our patients.
Encourage participation		12	My supervisor seriously considers staff suggestions for improving patient safety.
		13	My supervisor encourages me to express my ideas and suggestions regarding patient safety improvement.
		14	My supervisor encourages us to take initiative on improving patient safety whenever it is possible.
	Creation of safety awareness	15	We are given feedback about any changes about patient safety using an incident report.
		16	In this department, we discuss ways to prevent errors from happening again.
Create safety awareness		17	In this department, performance indicators for patient safety (e.g., pressure ulcers, hospital-acquired infections) are discussed.
		18	We compare our patient outcomes with results of other departments, and results of this benchmark are discussed.

## Data Availability

The use of data in the other studies excluding this study is restricted by the Institutional Review Board and cannot be used publicly.
